# PF chemotherapy combined with a PD-1/CTLA-4 bispecific antibody as first-line treatment in patients with recurrent/metastatic head and neck squamous cell carcinoma: a prospective, single-arm, phase II clinical study

**DOI:** 10.3389/fonc.2026.1961360

**Published:** 2026-10-22

**Authors:** Wenjun Liao, Qiaohua Li, Yue Deng, Jun Zhang, Yue Zhao, Shichuan Zhang

**Affiliations:** 1 Department of Radiation Oncology, Radiation Oncology Key Laboratory of Sichuan Province, Sichuan Clinical Research Center for Cancer Sichuan Cancer Center, Sichuan Cancer Hospital & Institute, Affiliated Cancer Hospital of University of Electronic Science and Technology of China, Chengdu, China; 2 School of Medicine, University of Electronic Science and Technology of China, Chengdu, China

**Keywords:** cadonilimab, chemoimmunotherapy, head and neck squamous cell carcinoma, immunotherapy, PD-1/CTLA-4 bispecific antibody

## Abstract

**Purpose:**

This study evaluated the antitumor activity and safety of cadonilimab, a programmed cell death protein 1 (PD-1)/cytotoxic T-lymphocyte-associated protein 4 (CTLA-4) bispecific antibody, combined with platinum/carboplatin and 5-fluorouracil (PF) chemotherapy as first-line treatment for patients with recurrent or metastatic head and neck squamous cell carcinoma (R/M HNSCC).

**Methods:**

In this prospective, single-arm, phase II trial, patients with histopathologically confirmed R/M HNSCC received cadonilimab (10 mg/kg) plus PF chemotherapy every 3 weeks for 4–6 cycles, followed by maintenance cadonilimab. The primary endpoint was 1-year progression-free survival (PFS). A target sample size of 30 was planned, but the study was terminated prematurely after enrolling 16 patients due to slow accrual.

**Results:**

Sixteen patients were enrolled. At a median follow-up of 18.4 months (range, 4.6 - 39.6), the 1-year PFS and overall survival (OS) rates were 50.0% (95% CI: 30.6% - 81.6%) and 75.0% (95% CI: 56.5% - 99.5%), respectively. The Objective Response Rate (ORR) was 56.3% (95% CI: 29.9% - 80.2%). Median PFS and OS were 9.8 months (95% CI: 2.8 - NR) and 17.7 months (95% CI: 12.3 - NR), respectively. Grade ≥3 treatment-related adverse events (TRAEs) occurred in four patients (25%), with no treatment-related deaths or immune-related cardiac toxicities reported.

**Conclusion:**

In this exploratory study, first-line cadonilimab combined with PF chemotherapy exhibited potential antitumor activity and manageable safety profiles in patients with R/M HNSCC. Given the favorable baseline profile of the cohort and the single-arm design, these findings should be interpreted with caution. Larger randomized controlled trials are required to determine whether dual PD-1/CTLA-4 blockade offers an incremental benefit over standard anti-PD-1-based regimens.

## 1 Introduction

Head and neck squamous cell carcinoma (HNSCC) arises from the mucosal epithelium of the oral cavity, oropharynx, hypopharynx, and larynx (1, 2). Despite curative-intent local therapies, over 50% of patients with locally advanced disease eventually develop recurrent or metastatic (R/M) disease, which carries a poor prognosis, with median overall survival (mOS) ranging from 10 to 13 months and a 5-year OS rate below 40% (3, 4).

For nearly a decade, the EXTREME regimen—cetuximab plus platinum and 5-fluorouracil (5-FU)—remained the first-line standard for R/M HNSCC, yielding a median OS of approximately 10 months (5). The treatment landscape shifted with the introduction of immune checkpoint inhibitors (ICIs). The phase III KEYNOTE-048 trial established pembrolizumab—either as monotherapy (in programmed death-ligand 1 (PD-L1) high tumors) or in combination with chemotherapy—as a new standard of care, significantly improving OS compared with the EXTREME regimen (4). Nevertheless, the ORR in the combination arm was only 37%, and a substantial proportion of patients still experience primary resistance or rapid disease progression, underscoring the need for more effective frontline strategies (6).

Bispecific antibodies targeting complementary immune checkpoints represent a promising strategy to enhance antitumor immunity (7). The combination of CTLA-4 and PD-1 blockade has demonstrated synergistic efficacy across multiple metastatic solid tumors (8). Cadonilimab (AK104) is a first-in-class, tetravalent bispecific antibody that simultaneously blocks PD-1 and CTLA-4. Its structure eliminates Fcγ receptor binding, thereby reducing Fc-mediated effector functions and potentially improving the therapeutic index (9). In a phase Ib/II study, cadonilimab monotherapy achieved an ORR of 32.3% in patients with advanced solid tumors, with a rate of 14% for grade ≥3 immune-related adverse events (iRAEs) (10). In locally advanced HNSCC, neoadjuvant cadonilimab combined with chemotherapy demonstrated impressive activity, achieving an ORR of 83.3% and a major pathological response rate of 76.7% (11).

Despite these advances, prospective data on cadonilimab combined with chemotherapy as first-line treatment for R/M HNSCC are lacking. To address this gap, we conducted a single-arm, phase II trial to evaluate the safety and preliminary antitumor activity of cadonilimab plus PF chemotherapy in treatment-naïve patients with R/M HNSCC.

## 2 Materials and methods

### 2.1 Study design and participants

This was a prospective, single-arm, phase II trial conducted at Sichuan Cancer Hospital. The study protocol was approved by the Ethics Committee of Sichuan Cancer Hospital and conducted in accordance with the Declaration of Helsinki and Good Clinical Practice guidelines. The trial was registered with the China Clinical Trial Registry (ChiCTR2200067129). All patients provided written informed consent.

Eligible patients were aged ≥18 years with histologically confirmed R/M HNSCC (oral cavity, oropharynx, larynx, or hypopharynx) not amenable to curative surgery or radiotherapy. Key inclusion criteria included no prior systemic therapy for R/M disease, at least one measurable lesion per Response Evaluation Criteria in Solid Tumors (RECIST) version 1.1, Eastern Cooperative Oncology Group (ECOG) performance status 0 - 1, life expectancy ≥6 months, and adequate organ function. Key exclusion criteria included prior exposure to PD-1/PD-L1 or CTLA-4 inhibitors, active autoimmune disease, other malignancies within 2 years, systemic immunosuppression within 14 days, symptomatic central nervous system metastases, or active infection.

### 2.2 Procedures

Treatment consisted of an induction phase followed by a maintenance phase. During induction, patients received intravenous cadonilimab (10 mg/kg) on day 1 plus PF chemotherapy every 3 weeks for 4–6 cycles. The PF regimen consisted of cisplatin (80 mg/m² divided over days 1 - 3) or carboplatin [target area under the curve (AUC) of five on day 1], combined with 5-fluorouracil (1000 mg/m²/day as a continuous infusion over 4 days). Patients who did not experience disease progression or unacceptable toxicity proceeded to maintenance therapy with cadonilimab (10 mg/kg) every 3 weeks until disease progression, unacceptable toxicity, or withdrawal of consent.

### 2.3 Biomarker evaluation

In HNSCC, particularly oropharyngeal squamous cell carcinoma (OPSCC), p16 immunohistochemistry (IHC) serves as a surrogate marker for high-risk human papillomavirus (HR-HPV) infection. In accordance with the AJCC 8th edition staging manual and the College of American Pathologists (CAP) guidelines, p16 positivity was defined as strong and diffuse nuclear and cytoplasmic staining in ≥70% of tumor cells. PD-L1 expression was evaluated on formalin-fixed paraffin-embedded (FFPE) pre-treatment archival or fresh core-needle tumor biopsy specimens using the PD-L1 IHC 22C3 pharmDx assay on the Dako Autostainer Link 48 platform (Agilent Technologies, Carpinteria, CA, USA) in accordance with the manufacturer’s instructions. The Combined Positive Score (CPS) was calculated as the number of PD-L1-positive cells (membrane-stained tumor cells and membrane/cytoplasm-stained tumor-associated immune cells) divided by the total number of viable tumor cells, multiplied by 100 (maximum score: 100). Guided by the latest NCCN and CSCO guidelines, PD-L1 expression was categorized as negative (CPS < 1), positive (CPS ≥ 1), or high expression (CPS ≥ 20).

### 2.4 Study endpoints

The primary endpoint was 1-year PFS. Secondary endpoints included ORR, 1-year OS, and safety. Tumor responses were assessed every 6–9 weeks per RECIST v1.1. PFS was defined as the duration from enrollment to the first documented instance of disease progression or death from any cause. OS was defined as the time from enrollment to death from any cause. ORR was defined as the proportion of patients achieving a complete response (CR) or partial response (PR). Adverse events (AEs) were graded according to the National Cancer Institute Common Terminology Criteria for Adverse Events (NCI CTCAE) version 5.0.

### 2.5 Statistical analysis

This study aimed to preliminarily evaluate the efficacy of cadonilimab, with a plan to include 30 patients; however, a total of 16 patients were ultimately enrolled. All analyses were performed using R version 4.4.0. Survival outcomes were analyzed using the intention-to-treat (ITT) principle. Descriptive statistics were used for baseline characteristics, response rates, and AEs. PFS and OS were estimated using the Kaplan - Meier method. Figures were generated using GraphPad Prism 10 and R 4.4.0.

## 3 Results

### 3.1 Baseline characteristics and treatment exposure

From March 2023 to November 2024, a total of 16 patients were enrolled in this study. The baseline demographic and clinical characteristics of these patients are summarized in **Table 1**. The cohort had a median age of 59.5 years (range, 47 - 71) and was predominantly male (87.5%, n=14). The majority of patients (75.0%) presented with an excellent performance status (ECOG 0). Primary tumor sites were diverse, with the oral cavity (31.3%) and oropharynx (37.5%) being the most common, followed by the larynx (18.8%). P16 positivity was detected in 50.0% of patients with primary tumors in the oropharynx. Regarding PD-L1 expression, 37.5% of patients had a Combined Positive Score (CPS) of ≥20, 25.0% had a CPS of 1 - 19, and 31.3% had a CPS of <1, and data were missing for one patient (6.3%).

**Table 1:** Demographic and baseline clinical characteristics of the study cohort (N = 16).

Characteristics	Patients (n=16)
Median Age (Range; years)	59.5 (47 - 71)
Sex	
Male	14 (87.5%)
Female	2 (12.5%)
Smoker	
No	9 (56.3%)
Yes	7 (43.8%)
ECOG performance status score	
0	12 (75.0%)
1	4 (25.0%)
Disease classification at enrollment^a^	
Local Recurrence	8 (50.0%)
Regional Lymph Node Recurrence	6 (37.5%)
Distant Metastasis	4 (25.0%)
Bone	1 (6.3%)
Lung	3 (18.8%)
Primary tumor site	
Oral Cavity	5 (31.3%)
Oropharynx	6 (37.5%)
Larynx	3 (18.8%)
Hypopharynx	2 (12.5%)
P16 status	
Positive	3 (18.8%)
Oropharynx	3 (18.8%)
Negative	9 (56.3%)
Oral Cavity	4 (25.0%)
Oropharynx	3 (18.8%)
Hypopharynx	2 (12.5%)
NA	4 (25.0%)
CPS	
<1	5 (31.3%)
1 - 19	4 (25.0%)
≥20	6 (37.5%)
NA	1 (6.3%)

Data are presented as n (%) unless otherwise indicated.

^a^ Patients could present with both recurrence and metastasis at enrollment; therefore, the cumulative percentage in this category exceeds 100%.

ECOG, Eastern Cooperative Oncology Group; CPS, Combined Positive Score; NA, not available (or not assessed).

All 16 patients received the initial combination treatment phase, with a median of 5 cycles (range, 2 - 6). 6 patients (37.5%) discontinued treatment during this phase: 4 due to disease progression (PD) and 2 due to patient decision/treatment fatigue. 10 patients (62.5%) successfully transitioned to cadonilimab maintenance monotherapy. Of these 10 patients, 7 subsequently discontinued treatment due to PD, and 1 discontinued due to patient decision/treatment fatigue. The remaining 2 patients (12.5%) had an ongoing response and remained on maintenance cadonilimab at data cutoff.

### 3.2 Safety

All 16 patients were evaluable for safety and were included in the analysis. All patients experienced TRAEs, the majority were low-grade and manageable. Grade ≥3 TRAEs occurred in four patients (25%), and consisted primarily of hematologic toxicities: leukopenia (25.0%, n=4), neutropenia (18.8%, n=3), thrombocytopenia (12.5%, n=2), and anemia (6.3%, n=1), some patients experienced multiple concurrent grade ≥3 events. Regarding irAEs, hypothyroidism occurred in 7 patients (43.8%), all classified as grade 1 - 2 (grade 1, n=2; grade 2, n=5). Patients with grade 2 hypothyroidism were asymptomatic or mildly symptomatic and were successfully managed with oral levothyroxine replacement therapy, with no grade ≥3 irAEs observed. Notably, there were no cases of immune-related cardiac toxicity, no treatment-related deaths, and no discontinuations due to TRAEs (**Table 2**). No cases of immune-related colitis, hepatitis, pneumonia, dermatitis, or other irAEs were reported during the study.

**Table 2:** TRAEs during treatment with cadunilumab in combination with the PF regimen (N = 16).

TRAEs	Patients (n=16)		
	Grade 1–2	Grade 3	Grade 4
Leukopenia	9 (56.3%)	3 (18.8%)	1 (6.3%)
Neutropenia	7 (43.8%)	2 (12.5%)	1 (6.3%)
Thrombocytopenia	5 (31.3%)	1 (6.3%)	1 (6.3%)
Anemia	7 (43.8%)	1 (6.3%)	0
Hypothyroidism	7 (43.8%)	0	0
Immune-Related Cardio Toxicity	0	0	0
Elevated ALT	2 (12.5%)	0	0
Elevated AST	3 (18.8%)	0	0
Increased Creatinine	5 (31.3%)	0	0

Data are presented as n (%). Adverse events were graded according to the Common Terminology Criteria for Adverse Events (CTCAE), version 5.0.

TRAEs, Treatment-related Adverse Events; ALT, Alanine Aminotransferase; AST, Aspartate Aminotransferase.

### 3.3 Efficacy

All 16 patients were included in the efficacy analysis. The ORR was 56.3% (9/16; 95% CI: 29.9% - 80.2%), with all responding patients achieving a PR (56.3%; 95% CI: 29.9% - 80.2%). Stable disease (SD) was achieved in 31.3% (5/16; 95% CI: 11.0% - 58.7%) of patients, resulting in a disease control rate (DCR) of 87.5% (14/16; 95% CI: 61.7% - 98.4%). PD was observed in 12.5% (2/16; 95% CI: 1.6% - 38.3%) of patients. Treatment outcomes are detailed in **Table 3**.

**Table 3:** Best overall treatment response and clinical efficacy (N = 16).

Best overall response	Patients, n(%) (N=16)	95% CI (%)
CR	0	–
PR	9 (56.3)	29.9 - 80.2
SD	5 (31.3)	11.0 - 58.7
PD	2 (12.5)	1.6 - 38.3
ORR (CR + PR)	9 (56.3)	29.9 - 80.2
DCR (CR + PR + SD)	14 (87.5)	61.7 - 98.4

Data are presented as n (%) of patients. Tumor responses were evaluated according to the Response Evaluation Criteria in Solid Tumors (RECIST), version 1.1.

CR, complete response; PR, partial response; SD, stable disease; PD, progressive disease; ORR, objective response rate; DCR, disease control rate.

As of the data cut-off date of June 3, 2026, the median duration of follow-up was 18.4 months (range, 4.6 - 39.6). The 1-year PFS and OS rates were 50.0% (95% CI: 30.6% - 81.6%) and 75.0% (95% CI: 56.5% - 99.5%), respectively. As shown in **Figures 1A, B**, the median PFS for the entire cohort was 9.8 months (95% CI: 2.8 - NR), and the median OS was 17.7 months (95% CI: 12.3 - NR). Among the nine patients who achieved a PR, four patients (44.4%) maintained an ongoing response at the time of data cutoff. A total of eight patients (50.0%) were alive at the end of the follow-up period. Individual patient outcomes, including survival status, are detailed in the swimmer plot in **Figure 2**.

**Figure 1:**
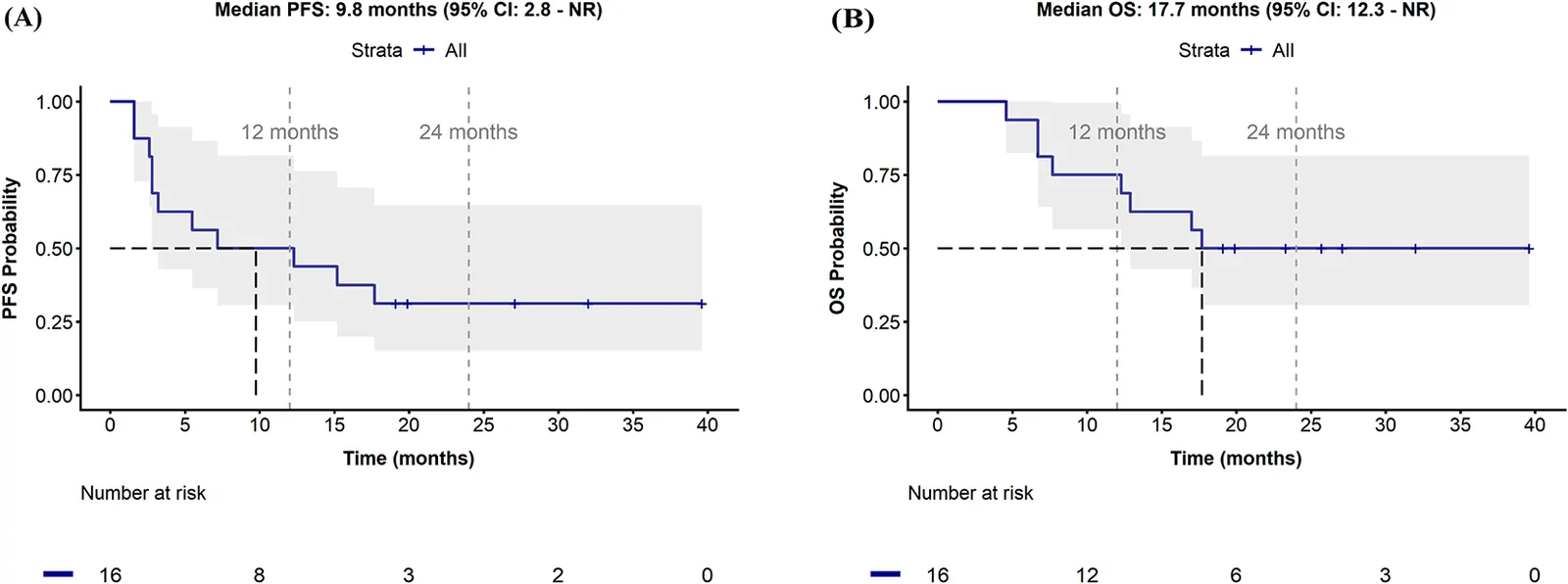
Kaplan-Meier estimates of survival in the study cohort (N = 16). **(A)** Kaplan-Meier curve for PFS. **(B)** Kaplan-Meier curve for OS. The shaded areas represent 95% confidence intervals (CI). Vertical tick marks (+) indicate censored data. The number of patients at risk at specified time points is displayed below the x-axis. Data are presented as median with 95% CI. PFS, progression-free survival; OS, overall survival.

**Figure 2:**
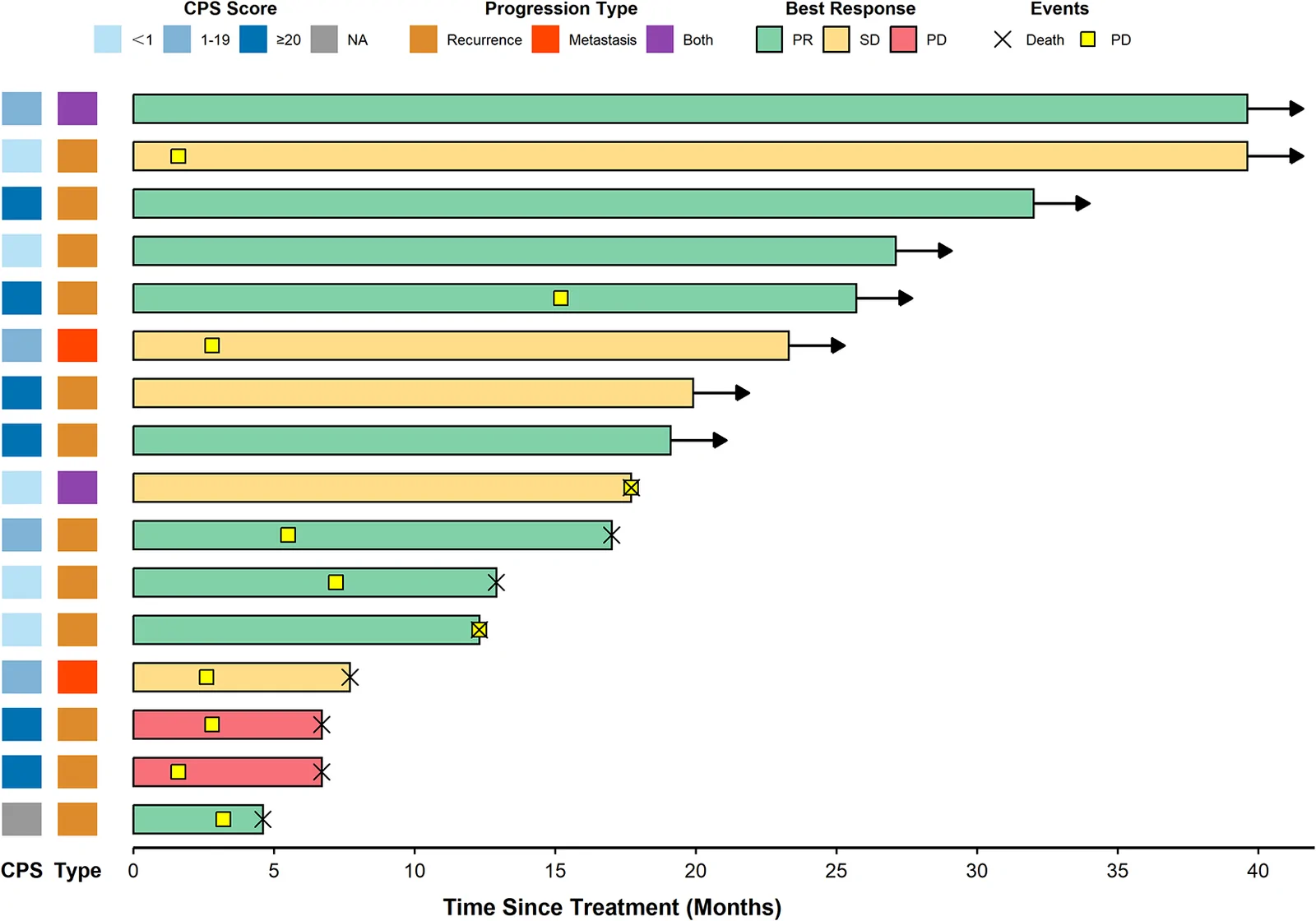
Swimmer plot of the clinical course and treatment outcomes. Each horizontal bar represents an individual patient, with the bar length indicating the duration from the initiation of treatment to the last follow-up or disease progression. The best objective response is color-coded as follows: PR (green), SD (yellow), and PD (red). Symbols denote specific events: cross, death; yellow square, progressive disease. Arrows indicate censored data for patients who remained on treatment or in follow-up at the time of the data cut-off. CPS, Combined Positive Score; NA, not available; PR, partial response; SD, stable disease; PD, progressive disease.

### 3.4 Exploratory biomarker analyses

Among the patients with a PD-L1 CPS ≥20 (n = 6), the ORR was 50.0% (3/6). In the group with CPS 1 - 19 (n=4), the ORR was also 50.0% (2/4), while those with CPS <1 (n=5) had an ORR of 60.0% (3/5). By p16 status, the ORR was 66.7% (2/3) in p16-positive patients and 44.4% (4/9) in p16-negative patients. Waterfall plot visualization suggested no significant differences in ORR were observed across subgroups with different levels of PD-L1 expression, and there was considerable overlap in the extent of tumor regression among the subgroups (**Figure 3**).

**Figure 3:**
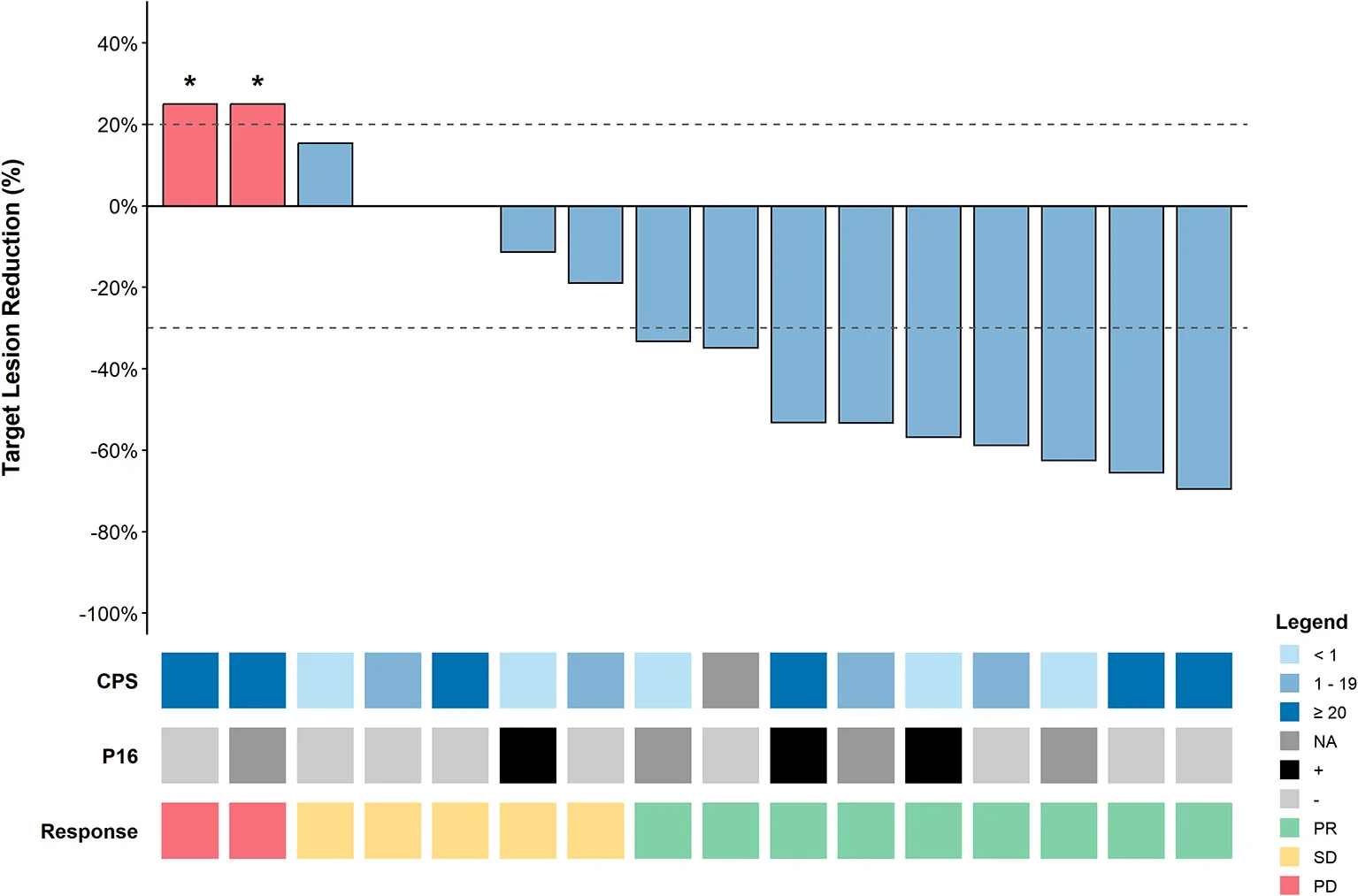
Waterfall plot illustrating the best percentage change from baseline in target lesion diameter. Each vertical bar represents an individual patient. The horizontal dashed lines represent the thresholds for PD (+20%) and PR (-30%) according to the Response Evaluation Criteria in Solid Tumors (RECIST) version 1.1. An asterisk (*) indicates patients whose post-treatment response was assessed as PD but for whom specific tumor growth data could not be measured. The heatmaps below the x-axis visualize the corresponding CPS, p16 status, and best overall response for each patient. CPS, Combined Positive Score; NA, not available; PR, partial response; SD, stable disease; PD, progressive disease.

A representative case of notable radiographic response is illustrated in **Figure 4**. Initially diagnosed in June 2022 with p16-positive, cT4aN2cM0 (AJCC 8th) oropharyngeal carcinoma (**Figure 4A**), the patient underwent induction chemotherapy with paclitaxel, cisplatin, and fluorouracil (TPF) followed by concurrent chemoradiotherapy (radiotherapy concurrent with cisplatin) between August 9 and November 28, 2022. After treatment, CR was achieved, and the disease was controlled for a duration of approximately 16 months, during which the patient underwent regular follow-up. However, during an MRI check on March 19, 2024, local recurrence of the tumor was detected (**Figure 4B**). The patient was subsequently enrolled in this study and received four cycles of cadonilimab in combination with chemotherapy. A follow-up MRI in July 2024 showed significant tumor shrinkage, with the treatment outcome assessed as a PR according to the RECIST v1.1 criteria (**Figure 4C**), and the duration of regression was 15.2 months.

**Figure 4:**
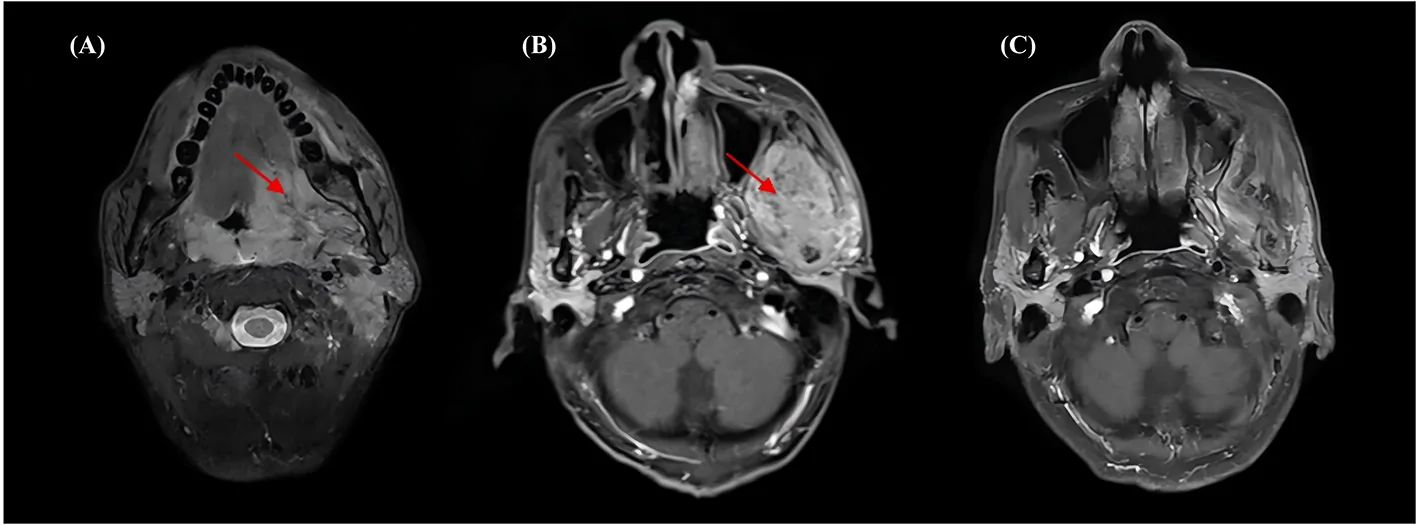
Magnetic Resonance Imaging (MRI) evaluation of a patient with recurrent oropharyngeal carcinoma undergoing chemoimmunotherapy. **(A)** Baseline imaging at initial diagnosis in early June 2022, showing locally advanced disease (cT4aN2cM0, red arrow). The patient subsequently underwent induction chemotherapy followed by radical chemoradiotherapy. **(B)** Recurrent lesion was identified on MRI in April 2024, 2 years after the completion of treatment. **(C)** MRI images in July 2024 after 4 cycles of cadunilumab combined with chemotherapy, showing marked regression of the primary lesion and achieving partial remission.

## 4 Discussion

In this exploratory phase II study evaluating cadonilimab plus PF chemotherapy as first-line treatment for patients with R/M HNSCC, the combination yielded an exploratory 1-year PFS rate of 50.0% (95% CI: 30.6% - 81.6%) at a median follow-up of 18.4 months (range, 4.6 - 39.6 months). The combination therapy yielded an ORR of 56.3% (95% CI: 29.9% - 80.2%). For the entire cohort, the median PFS was 9.8 months (95% CI: 2.8 - NR), and the median OS was 17.7 months (95% CI: 12.3 - NR), with a corresponding 1-year OS rate of 75.0% (95% CI: 56.5% - 99.5%). These efficacy outcomes compare favorably with historical data for dual immunotherapy or chemoimmunotherapy regimens in this setting. The findings support the potential of dual PD-1 and CTLA-4 blockade to enhance antitumor immunity, particularly when combined with chemotherapy. Nevertheless, cross-trial comparisons should be interpreted with caution, as our single-arm design does not permit direct assessment of the individual therapeutic contribution of the CTLA-4 component versus PD-1 inhibition alone.

The encouraging antitumor activity of cadonilimab may be attributed to its structurally optimized design. The elimination of Fcγ receptor binding abrogates Fc-mediated effector functions, including antibody-dependent cellular cytotoxicity (ADCC) and antibody-dependent cellular phagocytosis (ADCP), and is associated with a reduced risk of cytokine release-related toxicity (9). Mechanistically, PD-1 blockade restores the function of pre-existing tumor-specific T cells by reversing exhaustion within the tumor microenvironment (TME), whereas CTLA-4 blockade acts primarily during T-cell priming to promote the generation and expansion of novel T-cell clones. The concurrent blockade of both checkpoints exerts synergistic antitumor effects through complementary mechanisms: PD-1 inhibition reinvigorates and expands tumor-reactive T-cell populations already present in the TME, while CTLA-4 inhibition reduces immunosuppression by depleting regulatory T cells (Tregs). Together, these actions remodel the immunosuppressive TME, facilitating enhanced recruitment, infiltration, and sustained activation of tumor-infiltrating lymphocytes (TILs) (12).

Chemotherapeutic agents can induce immunogenic cell death (ICD) of tumor cells (13), triggering the release of damage-associated molecular patterns (DAMPs) and tumor antigens (14), which subsequently primes antigen-specific T-cell responses (15). Concurrently, certain chemotherapeutics can selectively deplete regulatory T cells (Tregs) and myeloid-derived suppressor cells (MDSCs), thereby mitigating the immunosuppressive tumor microenvironment (16), and converting immunologically “cold” tumors into “hot” tumors (17). From a clinical perspective, chemoimmunotherapy has demonstrated superior survival benefits compared with immunotherapy alone across various malignancies, including non-small cell lung cancer (NSCLC), triple-negative breast cancer (TNBC), and HNSCC (4, 18).

In the present study, the combination regimen yielded an ORR of 56.3%. In the KEYNOTE-048 trial, the pembrolizumab plus chemotherapy cohort reported an ORR of 36.0% (4). Notably, the current trial is limited by a small sample size and includes a patient population with favorable prognostic factors, such as p16-positive status. To outline the broader clinical landscape, **Table 4** summarizes various immunotherapy-based regimens. In the CheckMate 651 trial, nivolumab plus ipilimumab yielded an ORR of only 24.2%, with a median PFS of 3.3 months and a median OS of 13.9 months (19). Similarly, the KESTREL study reported ORRs of 17.2 - 21.8% for durvalumab with or without tremelimumab, with a median PFS of 2.8 months (20). In the LEAP-010 trial, the objective response rate for lenvatinib combined with pamlizumab was 46.1%, with a progression-free survival of 6.2 months (21). A Phase II study of camrelizumab combined with cetuximab and chemotherapy reported an ORR of 90% and a median PFS of 13.2 months (22).

**Table 4:** Summary of clinical efficacy of immunotherapy-based regimens in patients with R/M HNSCC.

Study name	Study population	Treatment intervention	mPFS (month)	mOS (month)	ORR (%)	Ref.
ChiCTR2200067129	Untreated R/M HNSCC	Cadonilimab + chemotherapy	9.8	17.7	56.3	
CheckMate 651	Untreated, incurable R/M HNSCC	Nivolumab + ipilimumab	3.3	13	24.2	(19)
KESTREL	Untreated, incurable R/M HNSCC	Durvalumab/ Durvalumab + tremelimumab	2.8/ 2.8	11.5/ 10.7	17.2/ 21.8	(20)
LEAP-010	R/M HNSCC	Pembrolizumab + lenvatinib	6.2	15.0	46.1	(21)
NCT05673577	R/M HNSCC	Camrelizumab + cetuximab + chemotherapy	13.2	Not reached	90.0	(22)
KEYNOTE-048	Untreated, incurable R/M HNSCC	pembrolizumab/ Pembrolizumab + chemotherapy	2.3/ 4.9	11.5/ 13.0	17.0/ 36.0	(4)
KEYNOTE-040	R/M HNSCC, postplatinum failure	pembrolizumab	2.1	8.4	Not reached	(23)
KEYNOTE-669	Untreated, incurable R/M HNSCC	pembrolizumab/ Pembrolizumab + epacadostat	–	–	21.0/ 31.0	(24)
CheckMate 141	R/M HNSCC, postplatinum failure	nivolumab	2.0	7.5	13.3	(25)
CheckMate 714	Untreated, incurable R/M HNSCC	Nivolumab + ipilimumab	Not reached	Not reached	13.2	(26)
HAWK	R/M HNSCC with PD-L1 ≥25%	durvalumab	2.1	7.1	16.2	(27)
EAGLE	R/M HNSCC, postplatinum failure	durvalumab/ Durvalumab + tremelimumab	2.1/2.0	7.6/6.5	17.9/18.2	(28)
CONDOR	R/M HNSCC with PD-L1<25%	durvalumab/ Durvalumab + tremelimumab	1.9/2.0	6.0/7.6	9.2/7.8	(29)

Cross-trial comparisons should be interpreted with caution due to inherent differences in patient selection, trial design, baseline characteristics, and standard of care practices.

R/M HNSCC, recurrent or metastatic head and neck squamous cell carcinoma; mPFS, median progression-free survival; mOS, median overall survival; ORR, objective response rate; Ref., references; -, not reported or not applicable.

Emerging data continue to expand the therapeutic landscape for R/M HNSCC. For instance, a randomized, double-blind phase III trial of finotonlimab combined with PF demonstrated a significant OS benefit over placebo plus chemotherapy (14.1 vs. 10.5 months) (30). Another phase II study of olaparib, pembrolizumab, and carboplatin reported an ORR of 51.7% and a median PFS of 7.8 months, with a median OS of 24.5 months (31). Additionally, the SAKK 11/16 trial of MVX-ONCO-1, a personalized cellular vaccine, showed clinical benefit in ICI-pretreated patients, with a 6-month OS rate of 68.8% (32). These studies highlight the diverse potential of immune-based combination strategies and provide context for our findings.

The safety profile observed in this study was generally manageable, with grade ≥3 treatment-related adverse events occurring in 25.0% of patients (4/16), primarily of hematological origin, including leukopenia, neutropenia, thrombocytopenia, and anemia. Notably, hypothyroidism was the only documented irAE (43.8%, 7/16). While the high incidence reflects the expected endocrine autoimmune reactivity of dual checkpoint inhibition, all cases were low grade (grade 1 - 2) and manageable with routine oral levothyroxine replacement without treatment interruption. No high-grade (grade ≥3) irAEs or classic toxicities such as immune-related colitis, pneumonitis, or hepatitis were observed. However, it is important to interpret these findings with caution, as the absence of these events may reflect the study’s limited sample size and follow-up duration rather than their true non-existence.

This study has several limitations. First, it is a single-center, phase II trial that was terminated prematurely, leading to a smaller-than-anticipated sample size (16 of the 30 planned patients). This smaller cohort may affect the generalizability of our findings and the robustness of the efficacy estimates, which might introduce a degree of selection bias. Second, the study cohort presented with favorable prognostic features, including good performance status (75.0% ECOG PS 0), a relatively high proportion of p16-positive oropharyngeal tumors (50.0%), and prevalent PD-L1 expression (CPS ≥1 in 62.5%), which limits the generalizability of these results to unselected, real-world R/M HNSCC populations. Third, because this was a single-arm study combining cadonilimab with chemotherapy without a direct anti-PD-1 monotherapy comparator, the precise therapeutic contribution and potential synergy attributable to the CTLA-4 blockade component remain difficult to pinpoint. Fourth, health-related quality of life (HRQoL) was not formally assessed. Finally, biomarker evaluations were limited to PD-L1 expression and p16 status, precluding a comprehensive exploration of other genomic or immunological correlates of response.

## 5 Conclusion

In this prospective single-arm phase II trial, first-line cadonilimab plus PF chemotherapy yielded encouraging signs of antitumor activity (1-year PFS rate 50.0%, 1-year OS rate 75.0%, ORR 56.3%) with an acceptable safety profile in patients with R/M HNSCC. Given the early study termination, resultant small cohort size, favorable baseline patient profile, and single-arm design, these findings cannot serve as a confirmatory endpoint evaluation and should be interpreted with caution. Further evaluation in adequately powered, randomized controlled trials with biomarker-driven patient stratification is warranted to define its clinical utility and determine whether dual PD-1/CTLA-4 blockade provides an incremental benefit over standard anti-PD-1-based regimens.

## Data Availability

The original contributions presented in the study are included in the article/supplementary material. Further inquiries can be directed to the corresponding author.

## References

[1] Vigneswaran N Williams MD . Epidemiologic trends in head and neck cancer and aids in diagnosis. Oral Maxillofac Surg Clin North Am. (2014) 26:123–41. doi: 10.1016/j.coms.2014.01.001 24794262 PMC4040236

[2] Gezer U Meral R Özgür E Yörüker EE Bronkhorst A Holdenrieder S . A narrative review of the role of non-viral circulating tumor DNA profiling in predicting the treatment response and recurrence in head and neck squamous cell carcinoma. Cancers (Basel). (2025) 17:2279. doi: 10.3390/cancers17142279 40723165 PMC12293434

[3] Vathiotis IA Johnson JM Argiris A . Enhancing programmed cell death protein 1 axis inhibition in head and neck squamous cell carcinoma: Combination immunotherapy. Cancer Treat Rev. (2021) 97:102192. doi: 10.1016/j.ctrv.2021.102192 33819755

[4] Burtness B Harrington KJ Greil R Soulières D Tahara M de Castro G Jr. . Pembrolizumab alone or with chemotherapy versus cetuximab with chemotherapy for recurrent or metastatic squamous cell carcinoma of the head and neck (KEYNOTE-048): a randomised, open-label, phase 3 study. Lancet. (2019) 394:1915–28. doi: 10.1016/s0140-6736(19)32591-7 31679945

[5] Vermorken JB Mesia R Rivera F Remenar E Kawecki A Rottey S . Platinum-based chemotherapy plus cetuximab in head and neck cancer. N Engl J Med. (2008) 359:1116–27. doi: 10.1056/NEJMoa0802656 18784101

[6] Tahara M Greil R Rischin D Harrington KJ Burtness B de Castro G . Pembrolizumab with or without chemotherapy in recurrent or metastatic head and neck squamous cell carcinoma: 5-year follow-up from the randomized phase III KEYNOTE-048 study. Eur J Cancer. (2025) 221:115395. doi: 10.1016/j.ejca.2025.115395 40262400

[7] Mardi A Aghebati-Maleki L . Bispecific antibodies (bsAbs) directed against PD-1/PD-L1 and CTLA-4; a mini review. Hum Antibodies. (2025) 33:3–9. doi: 10.1177/10932607251325751 40112316

[8] Park J Skålhegg BS . Combination of PD-1/PD-L1 and CTLA-4 inhibitors in the treatment of cancer - a brief update. Front Immunol. (2025) 16:1680838. doi: 10.3389/fimmu.2025.1680838 41159031 PMC12554671

[9] Pang X Huang Z Zhong T Zhang P Wang ZM Xia M . Cadonilimab, a tetravalent PD-1/CTLA-4 bispecific antibody with trans-binding and enhanced target binding avidity. MAbs. (2023) 15:2180794. doi: 10.1080/19420862.2023.2180794 36872527 PMC10012886

[10] Gao X Xu N Li Z Shen L Ji K Zheng Z . Safety and antitumour activity of cadonilimab, an anti-PD-1/CTLA-4 bispecific antibody, for patients with advanced solid tumours (COMPASSION-03): a multicentre, open-label, phase 1b/2 trial. Lancet Oncol. (2023) 24:1134–46. doi: 10.1016/s1470-2045(23)00411-4 37797632

[11] Cao F Li Y Fang Q Lin R Zhao Z Xu P . Cadonilimab (a PD-1/CTLA-4 bispecific antibody) plus neoadjuvant chemotherapy in locally advanced head and neck squamous cell carcinoma: A phase II clinical trial. Clin Cancer Res. (2025) 31:3876–85. doi: 10.1158/1078-0432.Ccr-25-1445 40705040

[12] Wang DR Wu XL Sun YL . Therapeutic targets and biomarkers of tumor immunotherapy: response versus non-response. Signal Transduct Target Ther. (2022) 7:331. doi: 10.1038/s41392-022-01136-2 36123348 PMC9485144

[13] Qi J Jin F Xu X Du Y . Combination cancer immunotherapy of nanoparticle-based immunogenic cell death inducers and immune checkpoint inhibitors. Int J Nanomedicine. (2021) 16:1435–56. doi: 10.2147/ijn.S285999 33654395 PMC7910111

[14] Krysko DV Garg AD Kaczmarek A Krysko O Agostinis P Vandenabeele P . Immunogenic cell death and DAMPs in cancer therapy. Nat Rev Cancer. (2012) 12:860–75. doi: 10.1038/nrc3380 23151605

[15] Sprooten J Laureano RS Vanmeerbeek I Govaerts J Naulaerts S Borras DM . Trial watch: chemotherapy-induced immunogenic cell death in oncology. Oncoimmunology. (2023) 12:2219591. doi: 10.1080/2162402x.2023.2219591 37284695 PMC10240992

[16] Vincent J Mignot G Chalmin F Ladoire S Bruchard M Chevriaux A . 5-Fluorouracil selectively kills tumor-associated myeloid-derived suppressor cells resulting in enhanced T cell-dependent antitumor immunity. Cancer Res. (2010) 70:3052–61. doi: 10.1158/0008-5472.Can-09-3690 20388795

[17] Muhammad S Li B Zhong W Siddiqui H Zhao Y Wang J . AI-enhanced synergistic chemo-immunotherapy: From mechanistic insights to clinical translation. Crit Rev Oncol Hematol. (2026) 217:105064. doi: 10.1016/j.critrevonc.2025.105064 41325797

[18] Hofheinz RD Lorenzen S Sebastian M Bölükbas S Fichtner- Loibl S . Preoperative immunotherapy in oncology. Dtsch Arztebl Int. (2026) 123:46–52. doi: 10.3238/arztebl.m2025.0209 41264644 PMC12980815

[19] Haddad RI Harrington K Tahara M Ferris RL Gillison M Fayette J . Nivolumab plus ipilimumab versus EXTREME regimen as first-line treatment for recurrent/metastatic squamous cell carcinoma of the head and neck: The final results of CheckMate 651. J Clin Oncol. (2023) 41:2166–80. doi: 10.1200/jco.22.00332 36473143 PMC10115555

[20] Psyrri A Fayette J Harrington K Gillison M Ahn MJ Takahashi S . Durvalumab with or without tremelimumab versus the EXTREME regimen as first-line treatment for recurrent or metastatic squamous cell carcinoma of the head and neck: KESTREL, a randomized, open-label, phase III study. Ann Oncol. (2023) 34:262–74. doi: 10.1016/j.annonc.2022.12.008 36535565

[21] Licitra L Tahara M Harrington K de Mendoza MOH Guo Y Aksoy S . Pembrolizumab with or without lenvatinib as first-line therapy for recurrent or metastatic head and neck squamous cell carcinoma (R/M HNSCC): Phase 3 LEAP-010 study. Int J Radiat Oncol Biol Phys. (2024) 118:e2–3. doi: 10.1016/j.ijrobp.2024.01.01642574925

[22] Ji D Sang Y Yang Y Chen G Liu X Guo Y . Camrelizumab combined with cetuximab and chemotherapy in recurrent or metastatic head and neck squamous cell carcinoma (R/M HNSCC): 1-year outcomes from the phase II trial. J Clin Oncol. (2025) 43:6030. doi: 10.1200/JCO.2025.43.16_suppl.603042852411

[23] Cohen EEW Soulières D Le Tourneau C Dinis J Licitra L Ahn MJ . Pembrolizumab versus methotrexate, docetaxel, or cetuximab for recurrent or metastatic head-and-neck squamous cell carcinoma (KEYNOTE-040): a randomised, open-label, phase 3 study. Lancet. (2019) 393:156–67. doi: 10.1016/s0140-6736(18)31999-8 30509740

[24] Cho BC Braña I Cirauqui B Aksoy S Couture F Hong RL . Pembrolizumab plus epacadostat in patients with recurrent/metastatic head and neck squamous cell carcinoma (KEYNOTE-669/ECHO-304): a phase 3, randomized, open-label study. BMC Cancer. (2024) 23:1254. doi: 10.1186/s12885-023-11316-0 39054467 PMC11270762

[25] Ferris RL Blumenschein G Jr. Fayette J Guigay J Colevas AD Licitra L . Nivolumab for recurrent squamous-cell carcinoma of the head and neck. N Engl J Med. (2016) 375:1856–67. doi: 10.1056/NEJMoa1602252 27718784 PMC5564292

[26] Harrington KJ Ferris RL Gillison M Tahara M Argiris A Fayette J . Efficacy and safety of nivolumab plus ipilimumab vs nivolumab alone for treatment of recurrent or metastatic squamous cell carcinoma of the head and neck: The phase 2 CheckMate 714 randomized clinical trial. JAMA Oncol. (2023) 9:779–89. doi: 10.1001/jamaoncol.2023.0147 37022706 PMC10080406

[27] Zandberg DP Algazi AP Jimeno A Good JS Fayette J Bouganim N . Durvalumab for recurrent or metastatic head and neck squamous cell carcinoma: Results from a single-arm, phase II study in patients with ≥25% tumour cell PD-L1 expression who have progressed on platinum-based chemotherapy. Eur J Cancer. (2019) 107:142–52. doi: 10.1016/j.ejca.2018.11.015 30576970

[28] Ferris RL Haddad R Even C Tahara M Dvorkin M Ciuleanu TE . Durvalumab with or without tremelimumab in patients with recurrent or metastatic head and neck squamous cell carcinoma: EAGLE, a randomized, open-label phase III study. Ann Oncol. (2020) 31:942–50. doi: 10.1016/j.annonc.2020.04.001 32294530

[29] Siu LL Even C Mesía R Remenar E Daste A Delord JP . Safety and efficacy of durvalumab with or without tremelimumab in patients with PD-L1-low/negative recurrent or metastatic HNSCC: The phase 2 CONDOR randomized clinical trial. JAMA Oncol. (2019) 5:195–203. doi: 10.1001/jamaoncol.2018.4628 30383184 PMC6439564

[30] Shi Y Guo W Wang W Wu Y Fang M Huang X . Finotonlimab with chemotherapy in recurrent or metastatic head and neck cancer: a randomized phase 3 trial. Nat Med. (2024) 30:2568–75. doi: 10.1038/s41591-024-03110-7 38942993

[31] Adkins D Ley JC Liu J Oppelt P . Olaparib, pembrolizumab, and carboplatin as first-line treatment of recurrent or metastatic head and neck squamous cell carcinoma: A phase II trial. JCO Oncol Adv. (2025) 2:e2500049. doi: 10.1200/oa-25-00049 40832450 PMC12360191

[32] Fernandez E Vernet R Urwyler M Von Rohr O Charrier E Belkouch MC . Overall survival of recurrent/metastatic head & neck squamous cell carcinoma patients progressing after ≥ 1 line of systemic therapy, treated with MVX-ONCO-1, a novel, first in class cell encapsulation-based immunotherapy: results of SAKK 11/16, a phase IIa trial. Exp Hematol Oncol. (2025) 14:113. doi: 10.1186/s40164-025-00703-x 40887652 PMC12398963

